# Identification of Resistance Genes and Response to Arsenic in *Rhodococcus aetherivorans* BCP1

**DOI:** 10.3389/fmicb.2019.00888

**Published:** 2019-05-07

**Authors:** Andrea Firrincieli, Alessandro Presentato, Giusi Favoino, Rosita Marabottini, Enrica Allevato, Silvia Rita Stazi, Giuseppe Scarascia Mugnozza, Antoine Harfouche, Maurizio Petruccioli, Raymond J. Turner, Davide Zannoni, Martina Cappelletti

**Affiliations:** ^1^Department for the Innovation in Biological Systems, Agro-Food and Forestry, University of Tuscia, Viterbo, Italy; ^2^Department of Biotechnology, University of Verona, Verona, Italy; ^3^Department of Biological Sciences, University of Calgary, Calgary, AB, Canada; ^4^Department of Pharmacy and Biotechnology, University of Bologna, Bologna, Italy

**Keywords:** arsenic resistance genes, arsenate reduction, *Rhodococcus*, *R. aetherivorans* BCP1, Actinobacteria

## Abstract

Arsenic (As) ranks among the priority metal(loid)s that are of public health concern. In the environment, arsenic is present in different forms, organic or inorganic, featured by various toxicity levels. Bacteria have developed different strategies to deal with this toxicity involving different resistance genetic determinants. Bacterial strains of *Rhodococcus* genus, and more in general Actinobacteria phylum, have the ability to cope with high concentrations of toxic metalloids, although little is known on the molecular and genetic bases of these metabolic features. Here we show that *Rhodococcus aetherivorans* BCP1, an extremophilic actinobacterial strain able to tolerate high concentrations of organic solvents and toxic metalloids, can grow in the presence of high concentrations of As(V) (up to 240 mM) under aerobic growth conditions using glucose as sole carbon and energy source. Notably, BCP1 cells improved their growth performance as well as their capacity of reducing As(V) into As(III) when the concentration of As(V) is within 30–100 mM As(V). Genomic analysis of BCP1 compared to other actinobacterial strains revealed the presence of three gene clusters responsible for organic and inorganic arsenic resistance. In particular, two adjacent and divergently oriented *ars* gene clusters include three arsenate reductase genes (*arsC*1/2/3) involved in resistance mechanisms against As(V). A sequence similarity network (SSN) and phylogenetic analysis of these arsenate reductase genes indicated that two of them (ArsC2/3) are functionally related to thioredoxin (Trx)/thioredoxin reductase (TrxR)-dependent class and one of them (ArsC1) to the mycothiol (MSH)/mycoredoxin (Mrx)-dependent class. A targeted transcriptomic analysis performed by RT-qPCR indicated that the arsenate reductase genes as well as other genes included in the *ars* gene cluster (possible regulator gene, *arsR*, and arsenite extrusion genes, *arsA, acr3*, and *arsD*) are transcriptionally induced when BCP1 cells were exposed to As(V) supplied at two different sub-lethal concentrations. This work provides for the first time insights into the arsenic resistance mechanisms of a *Rhodococcus* strain, revealing some of the unique metabolic requirements for the environmental persistence of this bacterial genus and its possible use in bioremediation procedures of toxic metal contaminated sites.

## Introduction

Arsenic is the most prevalent environmental toxic compound and ranks first on the U.S. Environmental Protection Agency’s Superfund List^1^. In the environment, the inorganic arsenic mainly exists under two forms, i.e., arsenite As(III) and arsenate As(V), the former being the most toxic due to its high reactivity against thiol-containing glutathione and to the thiol-groups of protein regulatory cysteines inhibiting important biochemical processes (Shen et al., 2013). On the other hand, arsenate is a phosphate analog and can alter biochemical pathways through the formation of arsenylated metabolites (Németi and Gregus, 2009; Gottesman and Mustaev, 2018).

Arsenic biotransformation occurs in association with energetic metabolism and/or arsenic resistance mechanisms (Páez-Espino et al., 2009). Pathways involved in arsenic resistance exist in all organisms, i.e., bacteria, fungi and plants, and the most common mechanisms include distinct classes of enzymes, known as arsenate reductases, which catalyze the biotransformation of As(V) to As(III). In bacteria, the enzymes reducing As(V) can be classified into respiratory or detoxifying reductases. Specifically, the respiratory arsenate reductases ArrAB (encoded by *arr* genes) are involved in the utilization of As(V) as a terminal electron acceptor in anaerobic respiration (Saltikov and Newman, 2003). On the other hand, three distinct functional classes of bacterial arsenite reductases (encoded by *arsC* genes) belong to the low-molecular weight phospho-tyrosine phosphatase (LMWP) protein family and are involved in resistance mechanisms and detoxification, being their catalytic activity linked to specific reducing systems: (i) the thioredoxin (Trx)/thioredoxin reductase (TrxR)-dependent class, (ii) the glutathione (GSH)/glutaredoxin (Grx)-coupled class, (iii) and the mycothiol (MSH)/mycoredoxin (Mrx)-dependent class (Páez-Espino et al., 2009). Until now, the latter reducing mechanism has only been described in Actinobacteria (Ordóñez et al., 2009) and among these, only *Corynebacterium glutamicum* has been studied in detail, despite the high abundance of diverse Actinobacteria genera in arsenic contaminated sites and their high resistance to toxic metals (Bankar and Nagaraja, 2018).

Once As(V) is converted into As(III), the trivalent form is extruded from the cell using either ArsB or Acr3. These two arsenite efflux proteins belong to two different protein superfamilies, being the first a member of ion transporter superfamily and the second a member of the bile/arsenite/riboflavin transporter (BART) superfamily (Prakash et al., 2003; Mansour et al., 2007). ArsB has 12 membrane-spanning segments and transports both As(III) and Sb(III) [showing high affinity for Sb(III)]. Acr3 has 8–10 membrane spanning segments, is highly selective for As(III), and is more widely distributed than ArsB members over bacteria, archaea, and eukaryotes (Fu et al., 2009). Both ArsB and Acr3 can act as proton exchanger with antiporter activity toward As(III) in the form of As(OH)_3_, the latter being the form of arsenite at neutral pH (Yang et al., 2015). Further, when *arsA* gene is present within the *ars* operon, the efflux activity of both ArsB and Acr3 can be associated to the activity of the ATPase subunit ArsA. The coupled ATP-hydrolyzing activity greatly enhances the As(III) extrusion (Fu et al., 2009, 2010).

Other pathways involved in bacterial arsenic resistance include the As(III) oxidation to As(V), using As(III) as electron donor through the respiratory arsenite oxidase activity (encoded by *aox* genes) (Silver and Phung, 2005). Additional genes associated with various *ars* clusters include a putative thioredoxin reductase required for As(V) reduction, which requires NADPH as reducing power (*arsT*), and two genes of unknown function that have weak homology to oxidoreductases (*arsO* and *arsH*) (Wang et al., 2006). On the other hand, ArsM, ArsP, and ArsJ are involved in the biotransformation of organic forms of arsenic (also named as organoarsenical compounds), mainly methyl-arsenite [MAs(III)]. In this regard, arsenic demethylation is considered a biodetoxification mechanism as it involves the conversion of MAs(III) into the less toxic forms methyl-arsenate [MAs(V)] (catalyzed by ArsH) or arsenite As(III) (catalyzed by ArsI) (Ben Fekih et al., 2018).

Strains of the *Rhodococcus* genus, belonging to the Mycolata group of Actinobacteria, are aerobic non-sporulating bacteria, which possess extremophilic features in relation to their ability to survive in the presence of high concentrations of toxic compounds, desiccation and osmotic stress, carbon starvation, UV irradiation, a wide range of temperatures (from 4 to 45°C) (Bej et al., 2000; Alvarez et al., 2004; LeBlanc et al., 2008; de Carvalho, 2012; Bequer Urbano et al., 2013; Cappelletti et al., 2016). Due to environmental robustness and wide biodegradation abilities, *Rhodococcus* strains are ideal microorganisms for bioremediation and industrial uses (Martínková et al., 2009). In particular, the possibility to use *Rhodococcus* strains for bioremediation strategies is associated to their capacity to tolerate and resist toxic metals, which are typically present in contaminated environments, often in association with organic pollutants, e.g., crude oil (Atagana, 2011). The resistance/robustness of members of this genus was mainly related to mycolic acids-containing wall and to their physiological adaptation strategies, i.e., cell membrane composition modification and intracellular inclusions formation (Alvarez et al., 2004; de Carvalho, 2012). On the other hand, the genetics and molecular mechanisms by which *Rhodococcus* strains resist toxic metals are largely unknown. In this context, the present work describes the capacity of *Rhodococcus aetherivorans* BCP1 (hereafter named BCP1) to resist arsenic along with the molecular analysis of the genetic determinants involved in arsenic response. BCP1 is a model alkanotrophic actinobacterium able to degrade hydrocarbons and chlorinated alkanes (Orro et al., 2015; Presentato et al., 2018a; Cappelletti et al., 2019) and to tolerate high amounts of tellurite and selenite (Cappelletti et al., 2016; Presentato et al., 2016, 2018b,c, 2019). Specifically, this work reports the resistance capacity of BCP1 toward As(V) and As(III) along with its ability to reduce As(V) intracellularly. A genome-wide analysis has been performed to identify the genetic determinants involved in inorganic and organic arsenic resistance in BCP1, also in comparison to other Actinobacteria. A targeted gene expression analysis has been conducted to evaluate the transcriptional induction of the *ars* genes in association to As(V) reduction.

## Materials and Methods

### Bacterial Cultures and Arsenic Minimal Inhibitory Concentration (MIC)

*Rhodococcus atherivorans* BCP1 (DSM 44980) was pre-cultured in Luria-Bertani (LB), before being washed twice with sterile saline solution (NaCl, 0.9 w/v) and being inoculated at an initial optical density (OD_600_) of 0.05 in 50 mL of M9 mineral medium (Jain et al., 2012) amended with glucose (0.2% w/v), as the sole source of carbon and energy, and variable concentrations of either arsenate As(V) or arsenite As(III). Cultures were grown at 30°C for 24 h on a rotary shaker at 150 rpm to determine the minimum inhibitory concentration (MIC) of As(III) and As(V), by counting viable cells on solid LB plates (agarose in LB, 15% w/v).

### Arsenate Speciation Assays

BCP1 cells grown in the presence of 6 or 33 mM As(V) were centrifuged, washed three times with ultrapure water, and freeze-dried. The cells were re-suspended in 25 mL of HNO_3_ (1% v/v) and incubated overnight, before been subject to microwave-assisted extraction, and filtered through 0.22 μm PVDF filters. The filtered solution was used for arsenic quantification. Arsenic was quantified by hydride generation technique along with a PerkinElmer Optima 8000 DV Inductively Coupled Plasma–Optical Emission Spectrometry (ICP-OES), adding to the samples an equal volume of a reducing solution composed by 5% w/v of potassium iodide and ascorbic acid, and hydrochloric acid 10%, and incubating for 1 h. As(III) was quantified by ICP-OES without adding the reducing agents to the samples while As(V) was quantified by subtracting As(III) to the total arsenic content. A calibration curve was made using a solution of arsenic trioxide at known concentrations. Statistical analysis was carried out in R v3.2.3, performing a *T*-test.

### *In silico* Analysis of Arsenic Resistance Genetic Determinants

The genome of *R. aetherivorans* BCP1 was analyzed using Pathways Tools v18.2 (Caspi et al., 2014) in combination with MetaCyc database in order to predict the *ars* genes involved in inorganic arsenic resistance. Further, BCP1 genome was compared against 333 complete genomes of strains belonging to Actinobacteria phylum using the “Function profile” tool of the Integrated Microbial Genomes (IMG) database (Markowitz et al., 2012), in order to detect the genes involved in arsenic resistance mechanisms. Specifically, a database of protein family functions (Pfam) identifier was created to identify proteins known to be involved in the resistance to organoarsenical (both pentavalent and trivalent) compounds and to inorganic arsenic. The Pfam identifiers used to build the database were: Pfam02374 [anion-transporting ATPase (ArsA)]; Pfam02040 [arsenical pump membrane protein (ArsB)]; Pfam03960 [arsenate reductase and related proteins, glutaredoxin family (ArsC)]; Pfam06953 [arsenical resistance operon trans-acting repressor (ArsD)]; Pfam01022 [As(III)-responsive transcriptional repressor (ArsR)]; Pfam00230 [major intrinsic protein family (MIP/AQP)]; Pfam13847 [Methyltransf_31 (ArsM)]; Pfam03773 [Predicted permease ArsP_1 (ArsP)]; Pfam01758 [Sodium Bile acid symporter family (Acr3)]; and Pfam00903 [Glyoxalase (ArsI)]; Pfam00085 [Thioredoxin (ArsX)]; Pfam07992 [Pyridine nucleotide-disulphide oxidoreductase (ArsT)]; Pfam00743 [Flavin-binding monooxygenase-like ArsO)]. For ArsJ, the organoarsenical efflux permease of *Pseudomonas aeruginosa* DK2 (WP_003109849.1) was used as reference protein (Chen et al., 2016). The only *ars* and related genes organized in clusters were considered in the bioinformatics analyses. Large-scale multiple sequence alignments of specific *ars* gene products (e.g., ArsR in Supplementary Table S3) were conducted using MEGA 7.0 (Kumar et al., 2016) with MUSCLE as alignment method with default parameters.

### Sequence Similarity Network (SSN) and Phylogenetic Analysis of ArsC Proteins in Actinobacteria

The predicted amino acid sequences of the three *arsC* genes identified in BCP1 genome were aligned with the complete genomes of Actinobacteria available in IMG database in order to retrieve the ArsC homologs present in representative actinobacterial strains. In order to construct the SSN, the ArsC significant hits were subsequently aligned “all against all” using BLASTP. *E*-values were converted in similarity values using the Markov Cluster Algorithm implemented in TribeMCL (Enright et al., 2002), and a similarity matrix was generated with SPCPS (Nepusz et al., 2010). The final network was visualized in Cytoscape (Shannon et al., 2003).

The phylogenetic tree was built using all ArsC amino acid sequences included in the SSN. The analysis was performed using MEGA 7.0^2^ (Kumar et al., 2016). Multiple-sequence alignment was performed using MUSCLE with default parameters; a phylogenetic tree was generated using the Maximum Likelihood method and the bootstrap test with 1000 replicates. The final tree was visualized and edited in FigTree v1.4.2.

### Reverse Transcriptase Quantitative PCR (qRT-PCR)

Total RNA was isolated from BCP1 cells grown (up to exponential phase, OD_600_ = 0.5) in 100 mL of M9 medium supplied with glucose (0.2% w/v) and with glucose supplied with 6 or 33 mM of As(V). RNA extraction and DNase I treatments were performed as described by Cappelletti et al. (2011, 2015). The RNA concentration, purity, and integrity were determined using a Nanodrop spectrometer 1000-ND. Reverse transcription (RT) of 500 ng RNA was performed using a RevertAid First Strand cDNA synthesis kit (Thermo Fisher Scientific) with random hexamer primers according to the manufacturer’s instructions. For quantitative PCR (RT-qPCR) analysis, cDNA was diluted 50-fold (vol/vol) in the amplification reaction with KAPA SYBR FAST qPCR Master Mix (Bio-Rad) and 200 nM (each) gene-specific primers (see Supplementary Table S1). The RT-qPCR procedure was carried out on a CFX96 PCR cycler (Bio-Rad) as follows: 3 min of pre-denaturing, 40 cycles of 95°C for 3 s, 60°C for 30 s, and 60°C for 20 s, followed by a melting curve stage from 65 to 95°C. RT-qPCR experiments were performed in triplicate and negative controls were performed by omitting the reverse transcriptase to check for RNA purity from genomic DNA. The expression levels of target genes were normalized to the expression level of the reference gene 16S rRNA, which was previously reported as a stable reference gene in a *Rhodococcus* strain (DeLorenzo and Moon, 2018). The fold changes for each target genes were obtained using the ΔΔCt method (Pfaffl, 2001) considering the growth on glucose as the reference condition. A Student’s one-sample *t*-test was used to evaluate whether the gene targets were differentially expressed between the two As(V) supplied (6 or 33 mM). Differences were considered significant at *P* < 0.05. RT-PCR experiments were also performed using each cDNA as template (1 μL from the 1/5 dilution) of PCR reactions (95°C for 2 min followed by 30 cycles of 95°C for 30 s, 57°C for 30 s, 72°C for 30 s, and final extension of 72°C for 7 min 95°C) with BioTaq DNA polymerase (BIOLINE) using the manufacturer’s instructions and primer pairs (see Supplementary Table S1) that annealed on adjacent genes in order to evaluate *ars* gene co-expression in operons. PCR products were visualized in a 0.7% (w/v) agarose gel after electrophoresis.

## Results

### MIC of As(V) and As(III) in *R. aetherivorans* BCP1

**FIGURE 1:**
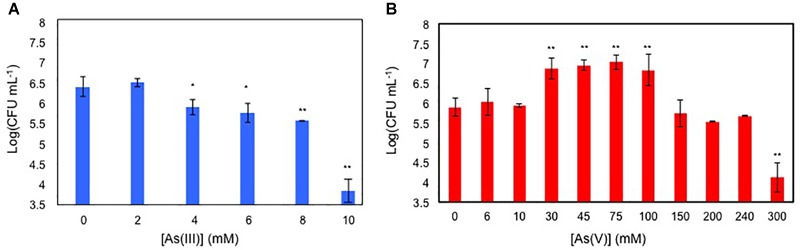
Viable cell counting, expressed as Log of CFU mL^-1^ of 24 h grown BCP1 cultures in the presence of increasing concentration of As(III) **(A)** and As(V) **(B)**. The resulting CFU/mL data were used to calculate the minimum inhibitory concentration (MIC) values of BCP1 toward As(III) and As(V). Mean values of three independent experiments with standard deviations are presented. Asterisks indicate that the cell counting is statistically different from that obtained in the absence of the oxyanion based upon Student’s test; ^∗^*P* < 0.05 and ^∗∗^*P* < 0.01.

Antimicrobial activity of As(V) and As(III) was assayed on glucose-growing BCP1 cells initially inoculated in M9 medium at Log (CFU mL^-1^) of 5 (Figure 1A,B). BCP1 cells grew up to 8 mM of As(III) (MIC_As(III)_ of 8 mM) and to 240 mM of As(V) (MIC_As(V)_ of 240 mM) (Figure 1A,B). Notably, cultures of BCP1 improved their growth performance when As(V) was added within a concentration range of 30–100 mM As(V) (Figure 1B). In particular, at 33 mM As(V), an improved growth performance was observed between 6 and 24 h of exposure to the oxyanion (Supplementary Figure S1).

### Arsenate Speciation in *R. aetherivorans* BCP1 Cells

**FIGURE 2:**
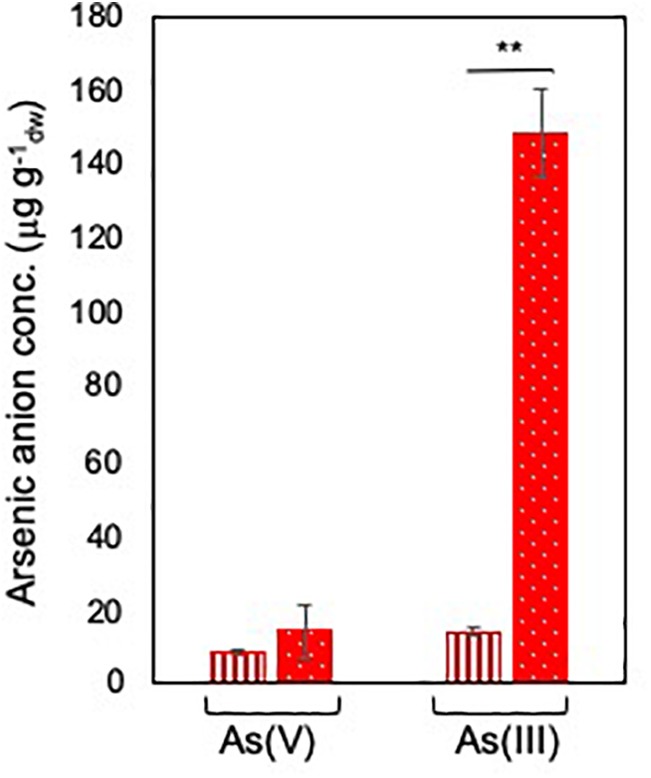
Arsenate speciation in *R. aetherivorans* BCP1 cells. Intracellular concentration of As(V) and As(III) (expressed as μg of arsenic per g of BCP1 cells measured as dry weight) as a function of 6 mM (vertical red stripes) and 33 mM (white dots on red background) As(V) supplied to the growth medium. Each experiment was performed in two technical triplicate sets. Asterisks indicate statistically significant difference based upon Student’s test; ^∗∗^*P* < 0.01.

Arsenate speciation was assessed by exposing BCP1 cells at sub-inhibitory concentrations of arsenate As(V), i.e., 6 and 33 mM in order to determine the capability of BCP1 to reduce As(V) into arsenite As(III) and to reveal if this process was dependent on the initial concentration of As(V). As a result, in response to different concentrations of As(V), no significant differences in the intracellular As(V) levels were observed, while the intracellular levels of As(III) were up to 10-fold higher when BCP1 cells were exposed to 33 mM of As(V) compared to 6 mM of As(V) (Figure 2). This clearly indicates that arsenate processing in BCP1 cells involves As(V) reduction to As(III).

### *In silico* Analysis of the Arsenic Resistance Genetic Determinants in BCP1 Genome and Comparison With Other Actinobacteria

**FIGURE 3:**
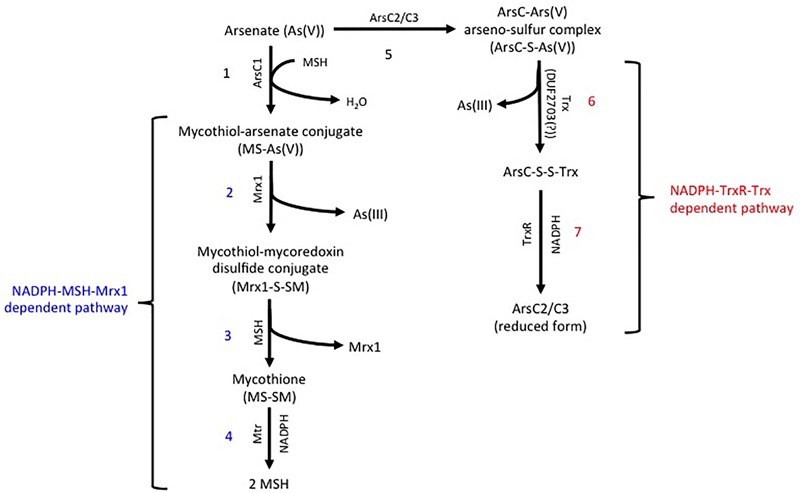
Predicted arsenic resistance pathway in *R. aetherivorans* BCP1 involving the NADPH-TrxR-Trx (indicated in red) and the NADPH-MSH-Mrx1 (indicated in blue) redox systems. In the MSH/ Mrx-dependent pathway (reactions from 1 to 4 on the left), a sulfide bond is generated between the As(V) and the thiol group of a cysteine in the MSH/ Mrx-dependent ArsC active site. MSH reduces the disulfide bond forming a mycothiol-arsenate conjugate [MS-As(V)] (reaction 1), which is reduced by mycoredoxin 1 (Mrx1) releasing As(III) and a mycothiol-mycoredoxin mixed disulfide conjugate (Mrx1-S-SM) (reaction 2). The mycothiol is recovered via MSH/MTR-pathway in which the disulfide bond Mrx1-S-SM is reduced by a second MSH (reaction 3), forming the mycothione (MS-SM) that is further reduced into mycothiols by the mycothione reductase (reaction 4). In the NADPH-TrxR-Trx pathway (reactions from 5 to 7 on the right), the sulfide bond between As(V) and the Trx-dependent ArsC proteins (reaction 5) is reduced through a thioredoxin system (possibly a DUF2703-containing protein in BCP1) with the release of As(III) (reaction 6). The reduction of the thiol-disulfide bond between the ArsC and Trx (ArsC-S-S-Trx) is catalyzed by a thioredoxin reductase (TrxR) (reaction 7). JGI locus tag and GenBank access codes: Mycoredoxin Mrx1 (Ga0035244_037036; N505_RS14145), ArsC1 (Ga0035244_05157; N505_RS21275), ArsC2 (Ga0035244_06161; N505_RS21290), ArsC3 (Ga0035244_05160; N505_RS21295), Mycothione reductase Mtr (Ga0035244_02642; N505_RS08670), DUF2703 (Ga0035244_05162; N505_RS21300), Thioredoxin reductase TrxR (Ga0035244_05792; N505_RS24495).

**FIGURE 4:**
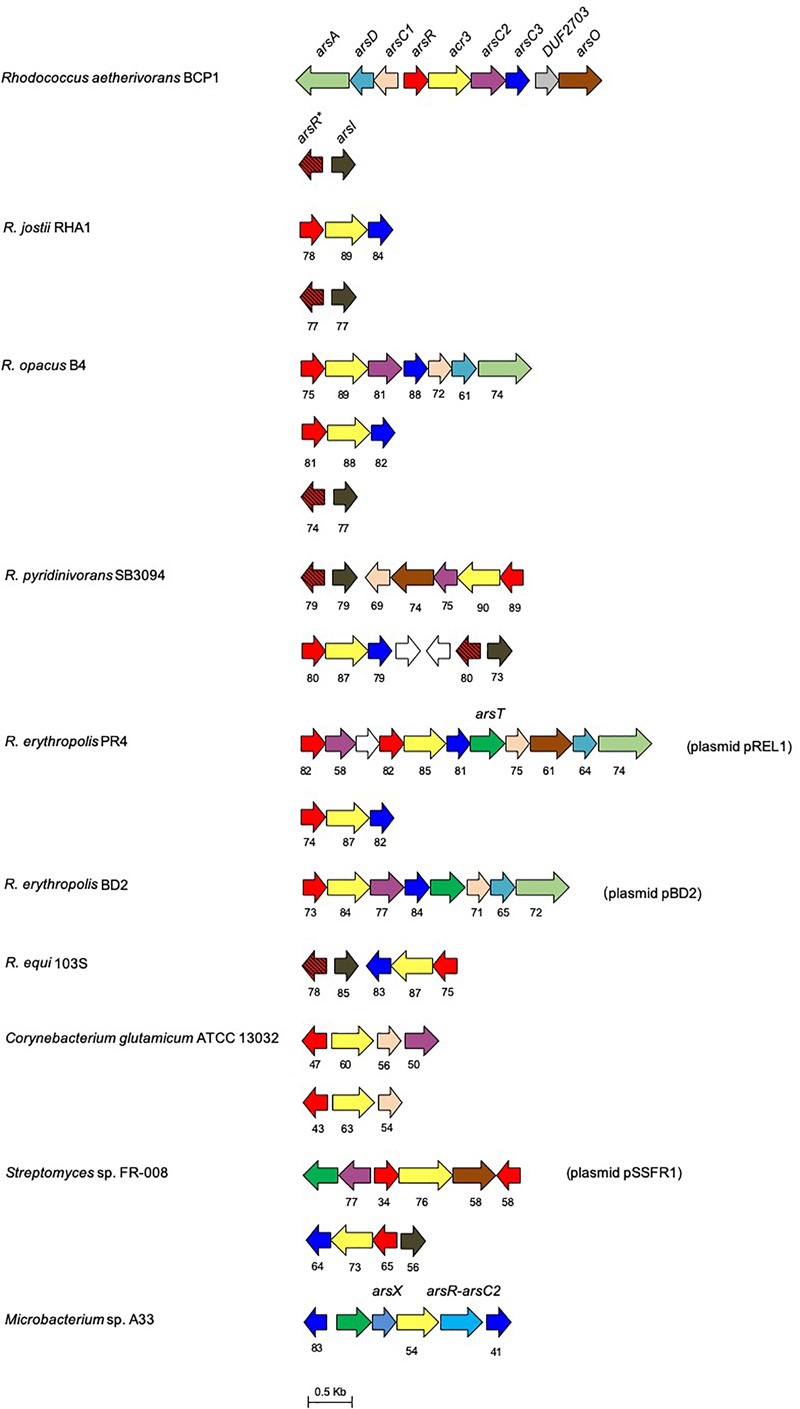
Organization of the *ars* gene clusters in *R. aetherivorans* BCP1 and other reference *Rhodococcus* and Actinobacteria strains. Genes are reported as arrows. Homologous genes are represented by the same color except for the white arrows that represent genes encoding hypothetical proteins. The % of aa identity of the gene products is shown below each *ars* gene compared to the corresponding homolog from BCP1. The *ars* genes product names are: ArsC1, mycothiol-arsenate transferase; ArsC2/3, thioredoxin-dependent arsenate reductase; ArsX, thioredoxin; ArsT, thioredoxin reductase; ArsR^∗^, MAs(III) responsive repressor; ArsR, As(III) responsive repressor; ArsO, arsenic resistance flavin-binding monooxygenase; Acr3, arsenical-resistance protein; ArsD, As(III) metallochaperone; ArsA, arsenic ABC transporter ATPase; ArsR-ArsC2, fusion protein between ArsR and ArsC2. The locus tags in GenBank and JGI are: *C. glutamicum* NCBI/JGI: cg1704-cg1707 and cg0317-cg0319, *R. ertythropolis* PR4 NCBI/JGI: RER_RS00775-00825 and RER_35220-35190, *Streptomyces sp.* FR-008 JGI: Ga0198356_117-112/NCBI: SFR_6896-6891 and JGI: Ga0198356_13214-13211/NCBI: SFR_3832-3835; *Microbacterium* sp. A33 only NCBI: AM283030; *R. aetherivorans* BCP1 JGI: Ga0035244_05155-05163/ NCBI: N505_RS21265- 21305 and JGI: Ga0035244_05182-0581/NCBI: N505_RS21405-RS21400, *R. equi* S103 NCBI/JGI: REQ_RS02250-RS02225 and REQ_04620-04560; *R. opacus* B4 NCBI/JGI: ROP_00150-00220, ROP_40650-40640 and ROP_29950-29970; *R*. *pyridinivorans SB3094* JGI*:* Rpyr3094_04678-04684/NCBI: Y013_RS25210-RS25175 and JGI: Rpyr3094_04607-04612/ NCBI: Y013_RS15395- RS15425; *R. jostii* RHA1 JGI: RHA1_ro03367-03369/NCBI: RHA1_RS16335-16345, JGI: RHA1_ro04133-ro04132/NCBI: RHA1_RS20100-RS20095.

In *R. aetherivorans* BCP1, the detoxification of inorganic arsenic was predicted to involve two homologs of the Trx-dependent ArsC (BCP1_ArsC2 and BCP1_ArsC3), and one homolog of the MSH/ Mrx-dependent ArsC through the use of PathwayTools (BCP1_ArsC1) (Figure 3). Members of these two ArsC classes are able to reduce As(V) to As(III) through a series of thiol/disulfide redox reactions, involving either the thioredoxin antioxidant system, i.e., NADPH-TrxR-Trx pathway (on the left, Figure 3), or the mycothiol redox system, i.e., NADPH-MSH-Mrx1 pathway (on the right, Figure 3; Li et al., 2007; Ordóñez et al., 2009). The BCP1 *arsC1, asrC2*, and *arsC3* genes are clustered with other *ars* genes within two flanked and divergently oriented transcriptional units (Figure 4). In particular, BCP1_*arsC2* and BCP1_*arsC3* (from here named as BCP1_*arsC2/3* when we refer to both) show an operon-like organization with the transcriptional regulator *arsR* gene and the arsenite transporter gene *acr3*, these latter two genes being overlapped by 4 bp (GTGA). A 121-nt long intergenic region separates *arsC3* from a gene encoding a DUF2703 domain featured by a CXXC thioredoxin redox motif, which is immediately followed by *arsO* gene that is predicted to encode a NAD(P)/FAD-dependent oxidoreductase. The BCP1 *arsO* is homolog to that described in *Streptomyces* sp. strain FR-008 as possibly involved in arsenic resistance (Wang et al., 2006), while DUF2703-domain proteins were categorized within a novel thioredoxin-fold family (Pawłowski et al., 2010) and in BCP1 a DUF2703-containing protein is likely involved in the thiol/disulfide redox reactions of the NADPH-TrxR-Trx pathway (Figure 3). The BCP1_*arsC1* gene belongs to the divergently oriented transcriptional unit, which comprises of an *arsA* gene encoding the arsenite transporting ATPase subunit and an *arsD* gene encoding the metallochaperone involved in delivering As(III) to ArsA.

**FIGURE 5:**
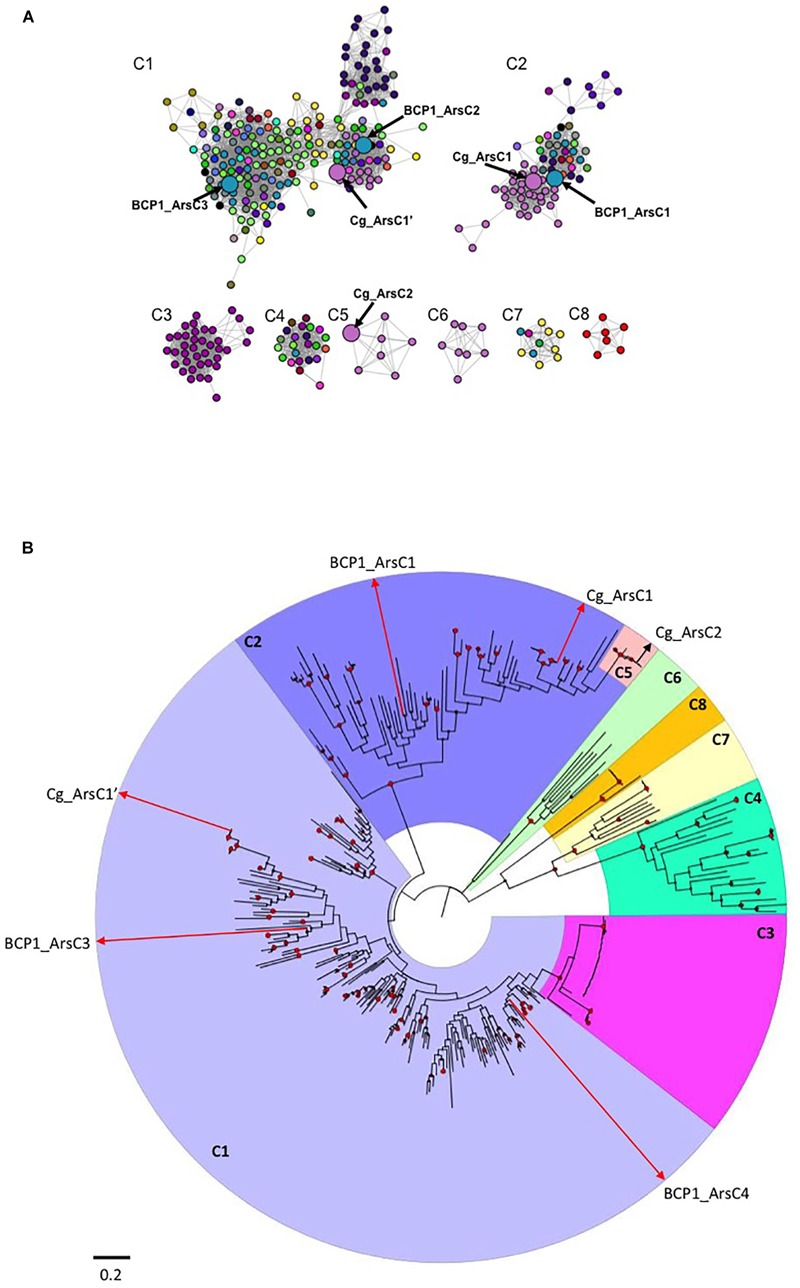
Sequence similarity network (SSN) and phylogenetic tree of ArsC proteins from Actinobacteria. In **(A)**, the SSN used a threshold for BLAST E-value of 1 × 10^-40^: only edges with E-values more significant than this thresohold value are included in the network. Differently colored nodes denote distinct bacterial genera. Enlarged nodes correspond to the specific *R. aetherivorans* and *C. glutamicum* arsenate reductases. The details on the ArsC proteins composing each cluster (C1–C8) and their taxonomy affiliations are provided in the file ArsC_SSN.cys in Supplementary Datasheet S3. In **(B)**, phylogenetic tree based on Actinobacteria ArsC amino acid sequences is shown. The tree was constructed by maximum likelihood method. Support values for each node were estimated using 1000 bootstrap. Nodes with support values higher than 70% are indicated with red circles. Each cluster from the phylogenetic tree is shaded with different color and indicated by the SSN corresponsing name (C1–C8). The raw data and details of the SSN and phylogenetic tree are provided in .cys and .nxs file, respectively, in Supplementary Datasheet S3.

An additional *ars* gene cluster is located 25 Kbp downstream of the *ars* cluster previously described and is composed by an *arsI* gene downstream of an *arsR* gene (Figure 5). This cluster is associated with the capability of BCP1 to detoxify organoarsenicals, being *arsI* gene product (a non-heme iron-dependent dioxygenase) involved in demethylation of methyl arsenite [MAs(III)] and dearylation of pentavalent aromatic arsenicals (Yoshinaga and Rosen, 2014). The multiple alignment of the BCP1 ArsI protein against homologs functionally characterized in *Bacillus* sp. MD1, *Thermomonospora curvata* DSM 43183, and *Nostoc* sp. PCC 7120, depicts the conservation of the cysteine pair Cys98-99 which is predicted to be the binding sites of MAs(III) and other trivalent organoarsenicals (Supplementary Figure S2) (Yoshinaga and Rosen, 2014; Yan et al., 2015; Nadar et al., 2016). In addition, the associated ArsR (named as ArsR^∗^) possesses two conserved cysteines corresponding to the cysteines 101 and 102 which represent the binding sites for the MAs(III) in the MAs(III)-responsive repressor ArsRs (Chen et al., 2017). Such features were generally conserved in all *Rhodococcus arsR* protein products detected in association to *ars*I genes (therefore named as *arsR^∗^* in Figure 4 and Supplementary Figure S3). Taken together, these results suggest that *Rhodococcus* strains, including BCP1, have retained the capability to respond and detoxify environmental MAs(III) through ArsI activity regulated by ArsR^∗^.

The organization of the *ars* gene clusters involved in BCP1 resistance to inorganic arsenic and organic arsenicals was compared against 333 genomes and 7 plasmids of Actinobacteria strains in order to evaluate the distribution of these resistance genetic determinants in other *Rhodococcus* and taxonomically related strains. As a result, the core of the *ars* genetic system in Actinobacteria included the *ars*R, *ars*C, and *acr*3 genes, these genes representing the basal resistance system to As(V) (Figure 4 and Supplementary Table S2). On the other hand, auxiliary genes encoding for the GlpF (glycerol uptake facilitator), ArsX (thioredoxin), ArsT (thioredoxin reductase), ArsA, ArsO, and ArsD were present only in a few actinobacterial genomes, with their distribution not being associated to any specific genus (Supplementary Table S2). Regarding *Rhodococcus* strains, all the genomes under analysis possessed an *arsO* gene, a half of them possessed *arsA* and *arsD*, and a few had also *arsT* (Figure 4 and Supplementary Table S2). Only in one actinobacterial genus, *Dermacoccus, ars*B was present in the *ars* gene cluster instead of *acr3* as gene coding for the arsenite efflux system. This is in line with the more widespread distribution previously reported for *acr3* over *arsB* (Fu et al., 2009). In regard of organic arsenicals, *arsI* was detected in clusters with other *ars* genes in the genomes of some species belonging to the genera *Actinoallotheicus, Amycolatopsis, Blastococcus, Frankia, Gordonia, Hoyosella, Kitococcus, Kuzneria, Mycobacterium, Mycolicibacterium, Nocardia, Rhodococcus, Streptomyces*, and *Tsukamurella*, and was the most abundant *ars* gene involved in the transformation of organic arsenic compounds (Supplementary Table S2). In general, actinobacterial *arsI* genes were either associated to the *arsR^∗^* gene encoding the MAs(III) responsive repressor ArsR^∗^ or they were included in *ars* clusters involved in inorganic arsenic resistance. In the latter case, the encoded ArsR proteins lacked the MAs(III)-binding signature composed by residues Cys101-102, indicating their specificity for As(III) rather than MAs(III) (Figure 4 and Supplementary Table S3).

Other genes known to be involved in the detoxification of arsenite methylated derivatives and pentavalent organoarsenicals are the ArsP transporter, the NADPH:FMN oxidoreductase ArsH, the S-adenosyl methionine methyltransferase ArsM, and the permease of the Major Facilitator Superfamily (MFS) ArsJ (Chen et al., 2016). ArsP, ArsH, and ArsM are involved in the resistance to MAs(III), being part of a newly discovered detoxification system that involves the direct extrusion of MAs(III) via ArsP, or the conversion of As(III) into MAs(III) by ArsM, and which is subsequently oxidized into MAs(V) by ArsH (Chen et al., 2015a,b). The genes *ars*P, *ars*J, *ars*H, and *arsM* are absent in *Rhodococcus* strains and they are generally rare in Actinobacteria. In particular, amongst the actinobacterial genomes and plasmids analyzed, *ars*J was identified in the *ars* gene cluster from an *Ilumatobacter* strain (*I. coccineum*) (Supplementary Table S2), nevertheless, similar genes encoding MFS proteins, which are functional analogs of ArsJ, were detected in *ars* gene clusters from *Amycolaptosys* and *Arthobacter* (data not shown). The gene *ars*H, which is involved in the conversion of MAs(III) to MAs(V) was also extremely rare, since it was identified only in one actinobacterial genus, *Rubrobacter* (Supplementary Table S2). In this genus, *arsH* was in cluster with *arsM* gene, suggesting the occurrence of resistance and detoxification mechanisms mediated by conversion of As(III) into MAs(V) via MAs(III) formation. In addition, *Rubrobacter arsH* gene shows high sequence similarity with the homolog described in *Pseudomonas* spp., suggesting horizontal gene transfer events (data not shown). The gene *arsM* has been detected in *ars* gene clusters, not including *arsH*, in several actinobacteria genomes under analysis. In particular, in *Cellulomonas gilvus, Amycolatopsys* strains, and a few strains of *Streptomyces, arsM* was detected in cluster with genes related to the resistance to inorganic arsenic and organoarsenical compounds. ArsM is known to convert As(III) into MAs(III), which is highly volatile but also highly toxic, and being previously associated to possible competitive advantage because of the antimicrobial effect (Yang and Rosen, 2016).

### Arsenate Reductases Protein Sequence Similarity Network

A protein sequence similarity network (SSN) and a phylogenetic tree were constructed using 357 actinobacterial ArsC proteins which were filtered on the basis of BLAST pairwise similarity score (i.e., *E* < 10^-40^) and cluster size (i.e., proteins included in clusters represented by less than 5 ArsC sequences were removed). Eight main groups were identified in the SSN, which were also supported by the phylogenetic analysis (Figure 5A,B and .cys and .nxs files in Supplementary Datasheet S3). The largest cluster found in SSN, named as C1, included three sub-clusters each representing thioredoxin (Trx)-dependent ArsC arsenate reductases (C1 in Figure 5A and .cys file in Supplementary Datasheet S3). C1 included the ArsC2 and ArsC3 of BCP1 along with the arsenate reductase Cg_ArsC1’ of *C. glutamicum* ATCC 13032, and Trx-coupled ArsC from *Streptomyces, Nocardia* and all the *Rhodococcus* strains under analysis (Figure 5A and .cys file in Supplementary Datasheet S3). The second SSN largest cluster C2 included the arsenate-mycothiol (MSH-coupled) transferase of *C. glutamicum* ATCC 13032 Cg_ArsC1 together with the homologs from other *Corynebacterium* species and from *Clavibacter, Mycolicibacterium* and *Rhodococcus* strains, including BCP1 (BCP1_ArsC1) (Figure 5A and .cys file in Supplementary Datasheet S3). The remaining clusters, C3-8, included either ArsC proteins from specific taxonomic groups (clusters C3, C5, C6, C8), or ArsC proteins fused with either an arsenite transporter ACR3 (C3) or a transcriptional repressor ArsR (clusters C4, C7). In particular, *C. glutamicum* ATCC 13032 Cg_ArsC2 was included in the small cluster C5 with arsenate-mycothiol transferases from only *Corynebacterium* strains. ArsC proteins from only *Bifidobacterium* species were grouped in cluster C8, while ACR3-ArsC fused proteins from only *Mycobacterium* and *Mycolicibacterium* species are included in C3.

The phylogenetic analysis supported the clusters resulting from the SSN analysis and also provided evolutionary relationship information on the 357 Actinobacteria ArsC proteins under analysis. In particular, the *Mycobacterium*-related Trx-dependent ArsC-Acr3 fused proteins of the cluster C3 are included within the C1 clade, and the *Corynebacterium*-related MHS-dependent ArsC proteins of C5 are included within the C2 clade (Figure 5B and .nxs file in Supplementary Datasheet S3). These diversification events occurred recently within each corresponding main clusters (the Trx-dependent C1 and the MSH-dependent C2, respectively). In particular, the fusion between an As(III) transporter Acr3 and an ArsC reductase occurred in the only *Mycobacterium* spp. strains (C3) during a fusion event against ArsC proteins from C1. In *Mycobacterium tuberculosis*, this fusion resulted in a single protein mediating both reduction and efflux processes (Wu et al., 2010). On the other hand, the Trx-dependent C8, including only *Bifidobacterium* ArsC, and C6, including ArsC1 from *Corynebacterium* species different from *C. glutamicum*, were phylogenetically unrelated with the other Trx-dependent clusters, the first one showing a completely separated evolutionary pathway and the second showing a common ancestor with C4 and C7, both composed by ArsC-ArsR fused proteins (Figure 5B and .nxs file in Supplementary Datasheet S3). Differently to Acr3-ArsC fused protein cluster C3, ArsR-ArsC fusion (in C4 and C7) was distributed among different genera and grouped separately from the other ArsC clusters.

From a functional point of view, the Trx-dependent Cg_ArsC1’ and the MSH-dependent Cg_ArsC1/2 from *C. glutamicum* are the only arsenate reductases characterized in Actinobacteria (Villadangos et al., 2011). In terms of sequence identity, BCP1_ArsC1/2/3 shared sequence similarity < 57% with the respective Cg homologs. However, the catalytic cysteines C88, C162 and C166 of Cg_ArsC1’ were conserved in BCP1_ArsC2 and BCP1_ArsC3 (Supplementary Figure S4A), and the amino acid residues C8, N11, V9, G12, G13, and K64 of active-site loop of Cg_ArsC2 were conserved in BCP1_ArsC1 (Supplementary Figure S4B), suggesting that BCP1_ArsC1/2/3 are catalytically active toward arsenate.

### Transcriptional Induction of *ars* Genes in *R. aetherivorans* BCP1

**FIGURE 6:**
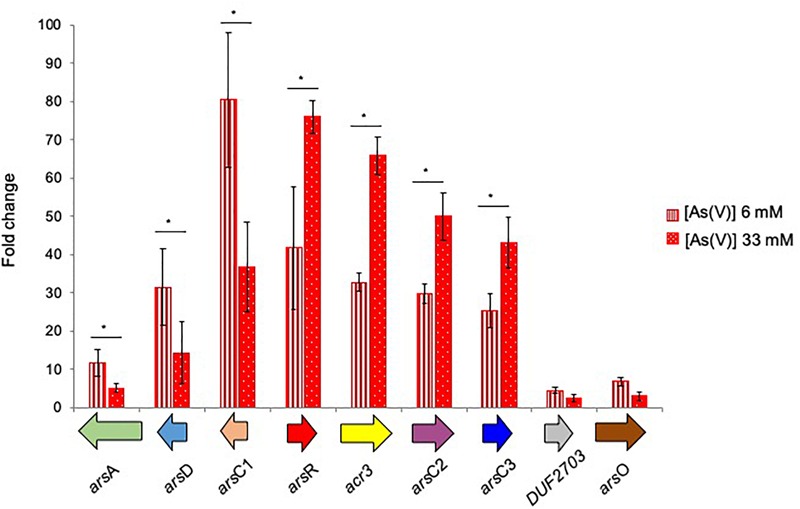
Fold changes in *ars* genes expression induced in *R. aetherivorans* BCP1 cells grown in the presence of 6 mM (vertical red stripes) and 33 mM (white dots on red background) As(V) assayed by RT-qPCR. Fold increases were calculated relative to the level of BCP1 cells grown on glucose (control). The housekeeping gene 16S rDNA was used as the reference gene to normalize the fold of expression of the target genes. Asterisks denote that the expression values obtained in the presence of the two As(V) concentration tested (6 and 33 mM) are statistically different based upon Student’s test (^∗^*P* < 0.05). Mean values of three independent experiments with standard deviations are presented.

The transcriptional induction of BCP1 *ars* genes was investigated in the presence of 6 and 33 mM of Ars(V) (Figure 6). All *ars* genes were transcriptionally induced by the presence of Ars(V) at different levels and their expression levels were also consistent with their organization in three operons, i.e., two divergently transcribed and one composed by *DUF2703* and *arsO* (Figure 6). In particular, higher concentration of As(V) induced higher expression of the operon *arsR-acr3-arsC2-arsC3*, while the opposite was seen for *arsC1-arsD-arsA*. The genes encoding a DUF2703-containing protein and the ArsO flavin-binding monooxygenase monooxygenase showed expression folds <10. Additional gene expression analyses using cDNA as template for PCR amplification with primers spanning adjacent genes, demonstrated the co-expression of consecutive *ars* genes within each transcriptional unit, showing that *DUF2703* and *arsO* were not co-transcribed with *arsC3* (Supplementary Figure S5).

Additional genes, possibly involved in arsenic resistance mechanisms such as those encoding the phosphate uptake system (*pst* genes) and those involved in mycothiol synthesis (*msh* genes), were not transcriptionally induced by As(V) under the culture conditions tested (Supplementary Figure S6 and Supplementary Table S4).

## Discussion

The alkanotrophic *R. aetherivorans* BCP1 strain is a polyextremophile since it tolerates high concentrations of organic solvents and resists toxic metalloids such as tellurite (TeO_3_^2-^) and selenite (SeO_3_^2-^) along with antibiotics and detergents (Cappelletti et al., 2016; Presentato et al., 2016, 2018a,b,c). One detoxification mechanism present in BCP1 cells is related to their capacity to convert toxic oxyanions into nanoparticles and nanorods (Presentato et al., 2018c). Here we show that electron dense nanostructures were also visible after the exposure of BCP1 cells to arsenate but the actual composition and structure of these precipitates has yet to be established (Supplementary Figure S7).

This study demonstrates that BCP1 strain is resistant to arsenic, with MIC of 8 mM for arsenite As(III) and 240 mM for As(V) (Figure 1), these values being similar to those reported in *Corynebacterium glutamicum* and in a few arsenic resistant *Rhodococcus* and other Actinobacteria strains isolated from contaminated sites (Mateos et al., 2006; Jain et al., 2012; Ghosh and Sar, 2013; Shagol et al., 2014; Maizel et al., 2018; Retamal-Morales et al., 2018). Further, BCP1 growth seems to benefit from exposure to As(V) 30–100 mM using glucose as sole carbon and energy source (Figure 1B). This observation shows some similarities with reports in other Actinobacteria (Bagade et al., 2016), an *Halomonas* strain (Wolfe-Simon et al., 2011), and two environmental isolates belonging to *Exiguobacterium* and *Aeromonas* genera (Anderson and Cook, 2004). Among the various proposals to explain the increase in cellular growth in the presence of a defined As(V) concentration range, we tested the one proposed by Anderson and Cook (2004), which is based on a pH variation due to a subsidiary (undefined) arsenate reduction mechanism. However, under our experimental conditions, no As(V) effect was found on the culture pH (data not shown). Taken together, these data suggest that further investigations are needed to unravel these interesting and still poorly understood metabolic aspect directly or indirectly related to As(V) reduction.

A genome-wide comparative analysis in Actinobacteria strains, including BCP1, revealed a great variety in the arsenic resistance genetic determinants among different *Rhodococcus* spp. and actinobacterial genera (Figure 4 and Supplementary Table S2). Generally, Actinobacteria genomes possess the only As(V) basic resistance genetic system represented by *arsR, acr3*, and *arsC* and, some of them also had the *arsI-arsR^∗^* genes involved in the resistance to organic forms of arsenic, i.e., MAs(III) and aromatic pentavalent arsenicals (being *arsR^∗^* distinguished from *arsR* because involved in organoarsenic resistance instead of inorganic arsenic compounds) (Yoshinaga and Rosen, 2014; Chen et al., 2017; Supplementary Table S2). *R. aetherivorans* BCP1 genome analysis revealed the presence of the *arsR^∗^-arsI* gene cluster and both the predicted ArsR^∗^ and ArsI products showed amino acid motifs required for binding of organosarsenical MAs(III) (Supplementary Figure S2). These results indicate the capacity of BCP1 to convert MAs(III) into As(III) as a detoxification pathway of the organoarsenicals. On the other hand, the genetic determinants involved in BCP1 resistance to inorganic arsenic are included in a composite genetic cluster constituted by two divergently oriented transcriptional units, i.e., *arsA-arsD-arsC1* and *arsR-acr3-arsC2-arsC3-DUF2703-arsO* (Figure 4). The co-localization of *arsR-acr3-arsC* with *arsD* and *arsA* genes was common among *Rhodococcus*, while most of Actinobacteria genomes did not have *arsA* and *arsD* (Figure 4 and Supplementary Table S2). The inorganic arsenic resistance gene cluster in BCP1 includes multiple *arsC* genes along with other genes associated to regulation (*arsR*) and transportation of As(III) (*acr3, arsD*, and *arsA*). The multiplicity of *arsC* genes in *ars* gene clusters is quite common among Actinobacteria strains and also among other *Rhodococcus* strains, except for the very well known *R. jostii* RHA1 and *R. equi* 103S, that possessed only one *arsC* gene in their genomes (Figure 4 and Supplementary Table S2). In particular, the three *arsC* genes of BCP1, named as *arsC1/2/3* genes, encode different classes of cytosolic arsenate reductases, namely: two members of the class of the thioredoxin (Trx)-dependent arsenate reductase (BCP1_ArsC2/3), and one member of the class of the MSH-dependent BCP1_ArsC1. The phylogenetic analysis and SSN of these arsenate reductases showed their clustering with ArsC paralogs present in other *Rhodococcus* and Actinobacteria strains (Figure 5). Among these, the arsenate reductases found in *Corynebacterium glutamicum* ATCC13032 are the only ArsCs functionally characterized (Messens et al., 2002; Villadangos et al., 2011). In particular, in *C. glutamicum*, the arsenic resistance system was reported to include the sequential activation of the arsenate reductases Cg_ArsC1’, Cg_ArsC1/2, whose enzymatic activities are based on the Trx/TrxR and MSH/Mrx systems, respectively (Villadangos et al., 2011). In this organism Cg_ArsC1’ was constitutively expressed and it was involved in the conversion of As(V) into As(III) at low arsenate concentration. The accumulation of As(III) was shown to trigger the expression of Cg_ArsC1/2 arsenate reductases. Further, *C. glutamicum* possesses a permease Acr3 as As(III) extrusion system which is coupled to available electrochemical energy (Fu et al., 2009, 2010), while it lacks an ArsA encoding gene which would allow the arsenite translocation mediated by ATP hydrolysis (Ordóñez et al., 2009).

As compared to *C. glutamicum* ArsCs, inorganic arsenic resistance gene cluster in BCP1 includes two Cg_ArsC1’ paralogs, i.e., BCP1_ArsC2/3 and one Cg_ArsC2 paralog, i.e., BCP1_ArsC1 (Figure 5). The *arsC2/3* genes are consecutive, while the *arsC1* is adjacent to *arsD* and *arsA* in a different transcriptional unit, which is upstream of that including *arsC2/3* and divergently oriented. All these three *arsC* genes were found to be transcriptionally induced in BCP1 cells exposed to As(V) supplied at two different sub-lethal concentrations (6 and 33 mM) (Figure 6). This is consistent with the ability of BCP1 to resist to As(V) through an intracellular reduction mechanism with the result of converting it into As(III), which was found to increase in concentration when BCP1 cells were exposed to higher initial As(V) doses (Figure 2). The apparent differential expression of *arsC2* from *arsC1* and *arsC3* depending on the As(V) concentration supplied (Figure 6) also suggest different regulatory mechanisms underlying the expression of the MSH-dependent and the Trx-dependent ArsC. Unlike *C. glutamicum*, BCP1 possesses an *arsA* gene in addition to *acr3*. In particular, the genes *arsA, arsD* and *acr3* are all induced by the presence of As(V), which is indicative of the production of an ATPase ArsA-Acr3 pump which can efficiently extrude the intracellular arsenite accumulated by the activity of BCP1 arsenate reductases. In this system, the metallochaperone ArsD was proved to deliver As(III) to the ATPase subunit ArsA supporting the As(III) extrusion system (Lin et al., 2007).

Genes coding for proteins involved in mycothiol production (*msh*D gene) and phosphate transport (*pst* genes) were clustered in BCP1 chromosome, suggesting a relation between mycothiol synthesis and the assembly and control of the inducible phosphate transporter Pst (Supplementary Figure S6). In this regard, a link between the production of mycothiol and arsenate resistance was demonstrated in *C. glutamicum* (Ordóñez et al., 2009). However, under the tested growth conditions, our results did not indicate *pstB, pstC, mshD* genes being transcriptionally induced by As(V) in BCP1 cells (Supplementary Table S4); on the contrary, their expression was negatively affected by the presence of inorganic arsenic under the tested growth conditions. Possible explanations for the lack of *pstB, pstC*, and *mhsD* induction include (i) the absence of phosphate starvation, (ii) the repression of phosphate transport genes as a way to prevent further acquisition of arsenate, (iii) the possible involvement of intermediate thiols derived from the mycothiol biosynthetic pathway instead of mycothiol (Newton et al., 2005; Sánchez-Riego et al., 2014).

## Conclusion

In conclusion, arsenate resistance in *R. aetherivorans* BCP1 is associated with (i) an apparent growth performance increase in the presence of As(V) at a defined concentration range, (ii) the reduction of As(V) into As(III), and (iii) the induction of *ars* genes involved in arsenate reduction, arsenite extrusion and transcriptional regulation, which are organized in two divergently transcribed operons. Bioinformatic analyses, performed to compare the genes included in *ars* operons of BCP1 and other actinobacterial strains detected different ArsC groups, which are indicative of different enzymatic activity and regulation. We believe that this work provides novel molecular and bioinformatics insights on the still poorly described arsenic resistance mechanisms occurring in *Rhodococcus* genus, and more in general, in Actinobacteria phylum.

## Author Contributions

AF carried out most of the experiments, participated in drafting the manuscript and along with MC conceived the experimental plan. MC took the lead in writing the manuscript and wrote the final version of it. Further, MC supervised the work and along with GF carried out the gene expression analyses by RT-qPCR. MP and AP helped planning the experiments and critically revised the manuscript along with GS and AH. AP also performed TEM analyses of BCP1 cells exposed to As(V). RM, SS, and EA supported AF in the arsenate speciation experiments and data interpretation. DZ and RT provided critical feedback and helped shape the manuscript. All authors contributed to manuscript revision, read and approved the submitted version.

## Conflict of Interest Statement

The authors declare that the research was conducted in the absence of any commercial or financial relationships that could be construed as a potential conflict of interest.

## Footnotes

1 http://www.atsdr.cdc.gov/spl/

2 http://www.megasoftware.net/
