# Supplementary material for: Identification of Resistance Genes and Response to Arsenic in Rhodococcus aetherivorans BCP1
Source: Front Microbiol. 2019 May 7;10:888. doi: 10.3389/fmicb.2019.00888 (PMC6514093; doi:10.3389/fmicb.2019.00888)
Supplement: Supplementary file 1 [file Data_Sheet_1.pdf]

Supplementary Material

Identification of resistance genes and response to arsenic in *Rhodococcus aetherivorans* BCP1

A. Firrincieli[1*], A. Presentato[2,3], G. Favoino[4], R. Marabottini[1], E. Allevato[1], S.R. Stazi[1], G. Scarascia Mugnozza[1], A. Harfouche[1], M. Petruccioli[1], D. Zannoni[4], R.J. Turner[3] and M. Cappelletti[4*]

[1]Department for the Innovation in Biological Systems, Agro-Food and Forestry (DIBAF), University of Tuscia (Italy);
[2]Department of Biotechnology, University of Verona (Italy)
[3]Department of Biological Sciences, University of Calgary (Canada);
[4]Department of Pharmacy and Biotechnology, University of Bologna (Italy)

Correspondence to

Dr. Martina Cappelletti
University of Bologna
Department of Pharmacy and BioTechnology
Via Irnerio 42, 40126
Bologna, Italy
e-mail: martina.cappelletti2@unibo.it

Dr Andrea Firrincieli
University La Tuscia
Department for the Innovation in Biological Systems, Agro-Food and Forestry (DIBAF), University of Tuscia (Italy)
Via Camillo de Lellis
Viterbo Italy
e-mail:andres.firrincieli@gmail.com

Table S1. Primer sets used in this study.

Target gene	Primer name	Sequence (5' to 3')
arsA	arsA-RT-For	AAGAAGGTGCTGCTGGTCTC
	arsA-RT-Rev	GACGATGCGTTCACGGTAGG
arsD	arsD-RT-For	ATGAGCACCATCGCAGTCTT
	arsD-RT-Rev	CCCTGATCCTTCAGCCACTG
arsC1	arsC1-RT-For	CTCAACGCCCTCTCGGTG
	arsC1-RT-Rev	GTGTCCCAGTTCTCCACGTA
arsR	arsR-RT-For	GACCATCTCGCACCATCTGA
	arsR-RT-Rev	GATCCCGGATTCGACGGAC
arsC2	arsC1'-RT-For	CCGAACTCGGACTCGATCTC
	arsC1'-RT-Rev	ACCACCTCGAGGGATTGTCC
arsC3	arsC1_1-RT-For	GACATCTCCACCCAATCCCC
	arsC1_1-RT-Rev	CCAGTCGCGGTAGCTCTTG
acr3	acr3-RT-For	CGCCATGGTCATCATCTGGA
	acr3-RT-Rev	GTGGTCTGTTCGAGTCCGAG
duf2703	duf2703-For	CGAATCCAACCGCATCTGG
	duf27o3-Rev	TTGACGATCAGTCGTTCGGG
arsO	arsO-RT-For	GTGACATCGTGATGGTCCCG
	arsO-RT-Rev	CGGTGTCGTCCCAGAAGATG
pstB	pstB-RT-For	CAATCCGTTCCCCACCATGT
	pstB-RT-Rev	CCTTGACCTCGTTCCACAGG
pstC	pstC-RT-For	GAGGCCACGATTACCACTCC
	pstC-RT-Rev	ATCACCGAGATGAGAACGGC
mshD	mshD-RT-For	GAACTGCTGCGGGTCAATG
	mshD-RT-Rev	ACTTTCGTCCAGTGGAAGCC

Table S2. Functional profile of Actinobacteria *ars* genes associated to *ars* gene clusters[a,b]

Actinobacterial Genus	# genomes	*glpF*	*arsM*	*arsH*	*arsP*	*arsJ*	*arsC*[c]	*arsB*	*arcR3*	*arsA*	*arsD*	*arsO*	*arsT*	*arsX*	*arsR*[d]	*arsI*
Acidimicrobium	1	-	-	-	-	-	-	-	-	-	-	-	-	-	-	-
Acidipropionibacterium	1	-	-	-	-	-	1	-	-	1	1	-	-	-	1	-
Acidothermus	1	-	-	-	-	-	-	-	-	-	-	-	-	-	-	-
Actinoallotheicus	1	-	-	-	-	-	1	-	1	-	-	-	-	-	1	1
Actinobacteria	4	4	-	-	-	-	4	-	-	-	-	-	-	-	-	-
Actinoplanes	4	3	-	-	-	-	4	-	-	-	-	-	2	-	4	-
Actinotignum	1	-	-	-	-	-	-	-	-	-	-	-	-	-	-	-
Adlercreutzia	1	-	-	-	-	-	-	-	-	-	-	-	-	-	-	-
Amycolaptosys	7	1	5	-	-	-	6	-	6	-	-	1	-	-	6	2
Arcanobacterium	1	-	-	-	1	-	1	-	-	-	-	-	-	1	1	-
Arthrobacter	2	2	-	-	-	-	2	-	2	1	-	2	2	-	2	-
Atopobium	1	-	-	-	-	-	-	-	-	-	-	-	-	-	-	-
Beutenbergia	1	-	-	-	-	-	1	-	-	-	-	-	-	-	1	-
Bifidobacterium	38	-	-	-	1	-	1	-	1	-	-	-	-	-	1	-
Blastococcus	1	-	-	-	-	-	1	-	1	-	-	-	-	-	1	1
Brachibacterium	1	-	-	-	-	-	1	-	1	-	-	1	-	-	1	-
Brevibacterium	2	-	-	-	-	-	-	-	-	-	-	-	-	-	-	-
Catenurispora	1	1	-	-	-	-	1	-	-	-	-	-	-	-	1	-
Cellulomonas	3	1	3	-	3	-	3	-	3	-	-	-	3	-	3	-
Clavibacter	3	-	-	-	-	-	3	-	-	-	-	-	-	-	3	-
Conexybacter	1	-	1	-	-	-	1	-	1	-	-	-	-	-	1	-
Coriobacterium	1	-	-	-	-	-	-	-	-	-	-	-	-	-	-	-
Corynebacterium	63	-	-	-	3	-	63	-	63	1	1	-	-	-	63	-
Cutibacterium	12	-	-	-	-	-	-	-	-	-	-	-	-	-	-	-
Criptobacterium	1	-	-	-	-	-	-	-	-	-	-	-	-	-	-	-
Cutibacterium	12	-	-	-	-	-	-	-	-	1	1	-	-	-	10	-
Dermacococcus	1	-	-	-	-	-	1	1	1	-	-	-	-	-	1	-
Eggerthella	2	-	-	-	-	-	-	-	-	-	-	-	-	-	-	-
Frankia	5	5	-	-	-	-	5	-	5	-	-	-	-	-	5	5
Gardnerella	4	-	-	-	-	-	-	-	-	-	-	-	-	-	-	-
Geodermatophilus	1	1	-	-	-	-	1	-	1	-	-	-	-	-	1	-
Glutamibacter	1	-	-	-	-	-	1	-	1	-	-	-	-	-	1	-
Gordonia	3	-	-	-	-	-	3	-	3	-	-	-	-	-	3	2
Gordonibacter	1	-	-	-	-	-	1	-	1	-	-	-	-	-	1	-
Hoyosella	1	-	-	-	-	-	1	-	1	-	-	-	-	-	1	1
Illumatobacter	1	-	-	-	-	1	1	-	1	-	-	-	-	-	1	-
Intrasporangium	1	-	-	-	-	-	1	-	1	1	1	-	-	-	1	-
Isoptericola	1	-	-	-	-	-	1	-	1	-	-	-	-	-	1	-
Jonesia	1	-	-	-	-	-	-	-	-	-	-	-	-	-	-	-
Kinecoccus	1	-	-	-	-	-	1	-	1	-	-	-	-	-	1	-
Kitasatospora	1	-	-	-	-	-	1	-	1	-	-	-	-	-	1	-
Kytococcus	1	-	-	-	-	-	1	-	1	-	-	-	-	-	1	1

Kocuria	1	-	-	-	-	-	1	-	1	-	-	-	-	-	-	-
Kutzneria	1	-	-	-	-	-	1	-	1	-	-	-	-	-	1	1
Kribella	1	-	-	-	-	-	-	-	-	-	-	-	-	-	-	-
Leifsonia	2	-	-	-	-	-	-	-	-	-	-	-	-	-	-	-
Microbacteirum	1	-	-	-	-	-	-	-	-	-	-	-	-	-	-	-
Micrococcus	1	-	-	-	-	-	1	-	1	-	1	-	-	-	1	-
Microlunatus	1	1	1	-	-	-	1	-	1	-	-	-	1	-	1	-
Micromonospora	2	-	-	-	-	-	1	-	1	-	-	-	-	-	1	-
Mobiluncus	1	-	-	-	-	-	-	-	-	-	-	-	-	-	1	-
Modestobacter	1	-	-	-	-	-	-	-	-	-	-	-	-	-	1	-
Mycobacterium	54	-	-	-	-	-	42	-	42	-	-	-	-	-	42	52
Mycolicibacter	1	-	-	-	-	-		-		-	-	-	-	-	1	-
Mycolicibacterium	8	1	-	-	2	-	8	-	8	2	2	-	-	-	8	6
Nakamurella	1	-	-	-	1	-	1	-	-	-	-	-	-	-	1	-
Nocardia	4	-	-	-	-	-	3	-	3	-	-	-	-	-	3	3
Nocardioides	1	-	-		-		1		1	-	-	-	-	-	1	1
Nocardiopsis	2	-	-	-	-	-	2	-	2	-	-	-	-	-	2	2
Olsenella	1	-	-	-	-	-	1	-	1	-	-	-	-	-	1	-
Paenaarthrobacter	1	1	-	-	-	-	1	-	1	-	-	-	1	-	1	-
Propionibacterium	1	-	-	-	-	-	1	-	-	1	1	-	-	-	1	-
Pseudoarthrobacter	2	2	-	-	-	-	2	-	-	-	-	-	1	1	1	-
Pseudonocardia	1	-	-	-	-	-	1	-	1	-	-	-	-	-	1	1
Pseudopropionibacterium	1	-	-	-	-	-	-	-	-	-	-	-	-	-	-	-
Renibacterium	1	-	-	-	-	-	-	-	-	-	-	-	-	-	1	1
Rhodococcus	9	-	-	-	-	-	9	-	9	4	4	9	2	-	9	5
Rhodoluna	1	-	-	-	-	-	1	-	-	-	-	-	-	-	-	-
Rothia	2	-	-	-	-	-	-	-	-	2	2	-	-	-	2	-
Rubrobacter	3	-	2	2	-	-	3	-	3	-	-	-	-	-	3	-
Saccharomonospora	1	-	-	-	-	-	1	-	1	-	-	-	-	-	1	1
Saccharopolispora	1	-	-	-	-	-	1	-	1	-	-	-	-	-	1	1
Saccharotrix	1	-	-	-	-	-	-	-	-	-	-	-	-	-	1	-
Salinispora	2	1	-	-	-	-	2	-	2	-	-	-	-	-	1	1
Sanguibacter	1	-	-	-	-	-	1	-	1	-	-	-	1	-	1	-
Segniliparus	1	-	-	-	-	-	-	-	-	-	-	-	-	-	1	-
Slackia	1	-	-	-	-	-	-	-	-	-	-	-	-	-	1	-
Stackebrandtia	1	-	-	-	-	-	1	-	1	-	-	-	-	-	1	-
Streptomyces	25	3	5	-	-	-	19	-	19	-	-	-	2	-	19	15
Streptosporangium	1	-	-	-	-	-	1	-	1	-	-	-	-	-	1	-
Thermobifida	1	-	-	-	-	-	1	-	1	-	-	-	-	-	1	1
Thermobispora	1	-	-	-	-	-	-	-	-	-	-	-	-	-	1	1
Thermomonospora	1	-	-	-	-	-	-	-	-	-	-	-	-	-	1	1
Tropheryma	2	-	-	-	-	-	1	-	1	-	-	-	-	-	-	-
Trueperella	1	-	-	-	-	-	-	-	-	-	-	-	-	-	-	-
Tsukamurella	1	-	-	-	-	-	1	-	1	-	-	-	-	-	1	1
Verrucosispora	1	-	-	-	-	-	1	-	1	-	-	1	-	-	1	-

[a] The numbers reported in the table correspond to the number of actinobacterial strains possessing each gene organized in cluster with other genes involved in arsenic resistance
[b] The actinobacterial genomes and plasmids present in the JGI database with complete sequence have been included in the analysis
[c] These numbers include both thioredoxin dependent arsenate reductases (ArsC2/3) and mycothiol arsenate transferases (ArsC1)
[d] These numbers include both *arsR* and *arsR** predicted to regulate mechanisms of resistance to inorganic and organic arsenic compounds, respectively.

Table S4. Fold change of the *mshD*, *pstB* and *pstC* genes (see Supplementary Figure S6) in *R. aetherivorans* BCP1 cells exposed to 6 and 33 mM of As(V)[1,2]

Target genes	Gene Product	6 mM As(V)	33 mM As(V)
pstB	Phosphate transport system protein	2.01 ± 0.01	0.26 ± 0.05
pstC	Phosphate transport system permease protein	1.08 ± 0.01	0.25 ± 0.03
mshD	Mycothiol synthase	0.58 ± 0.03	0.78 ± 0.01

[1] Data reported are the fold of expression compared to the glucose-growth conditions without As(V) supplied to the medium, using 16S rRNA as reference gene.
[2] Data is presented as the mean ± SD (standard deviation) of three replicates

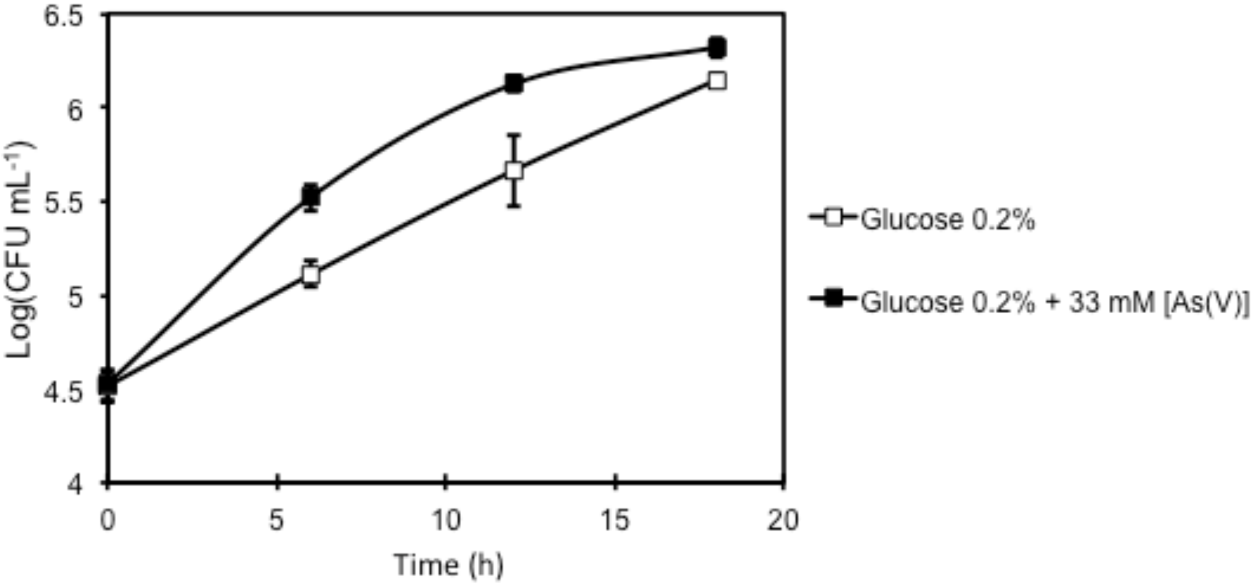

Figure S1 Growth curves of *R. aetherivorans* BCP1 without (white squares) and in the presence of 33 mM As(V) (black squares). Data are presented as the mean ± SD of five replicates.

```
BCP1_ArsI          --MSRVQLALNVDDLQEAVTFYSKLFGTEPAKVKPGYANFAIAEPPLKLVLIENA-GKG- 56
DSM43183_ArsI      --MSRVQLALRVPDLEASIGFYSKLFGTGPAKVRPGYANFAIAEPPLKLVLIEGA-GEDA 57
MD1_ArsI           --MKYAHVGLNVTNLEKSIEFYSKLFGAEPVKVKPDYAKFLLESPGLNFTLNLRDEV-NG 57
PCC7120_ArsI       MSVMKTHVALNVTNIEKSVTFYRAMFGLEPVKYKTDYAKFDIPNPALNLTLNLTNNVQIG 60
                     :  .::.*.* ::: :: **  :**  *.* : .**:* : .* *::.*

                                                         ▼▼
BCP1_ArsI          GSINHLGVEVESSEKVHSEIARLTDEGL-FTDEEIGTTCCFATQDKVWVTGPAGEKWEVY 115
DSM43183_ArsI      TRLDHLGVEVEDSAQVGHAARRLKESGL-ATVEENDTACCYAVQDKVWVTGPGGEPWEVY 116
MD1_ArsI           NQVGHFGIQVESTEEVVAHKNRLAENGILSQYDEINTTCCYALQDKFWIHDPDGNEWEFF 117
PCC7120_ArsI       GALSHLGVQVESTQEVQSAIERFNEAGL-DLFTEDNTDCCYALQDKVWVTDPDGNRWEVF 119
                     :.*:*::**.: :*      *: : *:     * .* **:* ***.*: .* *: **.:

BCP1_ArsI          TVLADSDTFGTSPKLL---DQGENSEGVCCGTAAEQEPATGTEKAPAATTCC-        164
DSM43183_ArsI      VVKGDADTLAKAD------------DSACCTPRD-----SGSAGAAVGADCC-        151
MD1_ArsI           YTKTTVEENSTHPPT-CCVNEPNVEKAECCSPTASSNK--------DTSNCCS        161
PCC7120_ArsI       VVKVADTAPEKNLATVSSSGEIQAVKKSCCA----------------------        150
                                               **
```

Figure S2 Multi-sequence alignment of the *R. aetherivorans* BCP1 As-C lyase ArsI against ArsI hortologs from *Bacillus* sp. MD1 (MD1_ArsI, AIA09488), *Thermomonospora* curvata DSM 43183 (DSM43183_ArsI, WP_012854466.1), and *Nostoc* sp. PCC 7120 (PCC 7120_ArsI, WP_010995277.1). Black triangle indicate the cysteine pair probably involved in MAs(III) binding. Sequence alignment was performed with Clustal Omega (https://www.ebi.ac.uk/Tools/msa/clustalo/)

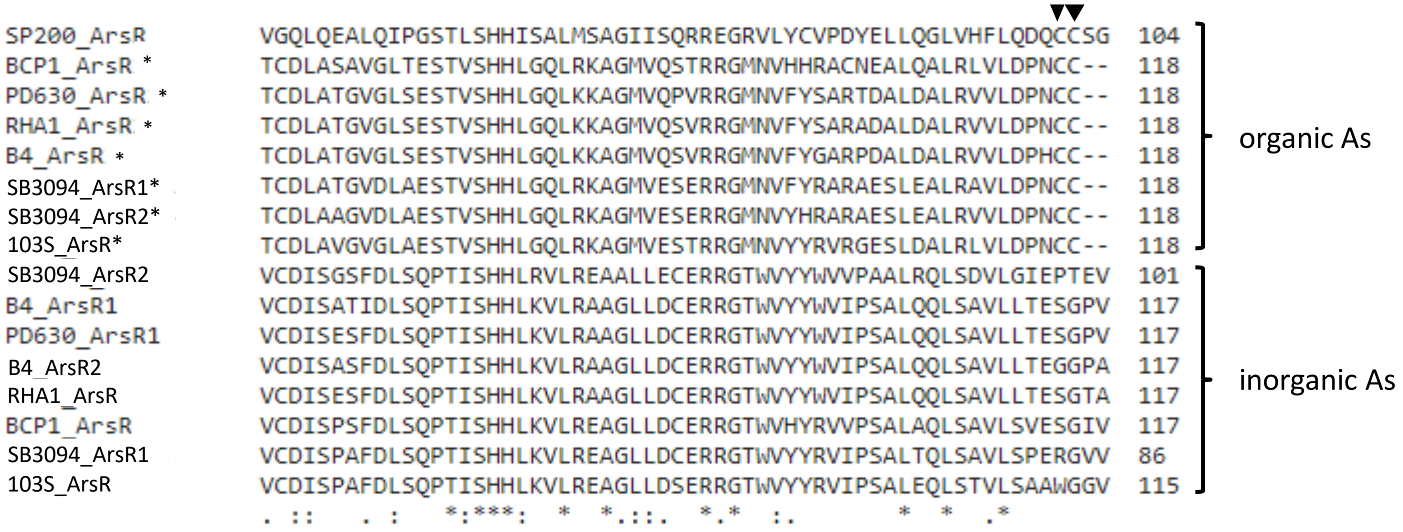

Figure S3 Multi-sequence alignment of the MAs(III)-responsive ArsR* transcriptional repressor of *Shewanella putrefacens* 200 (originally named as Sp200_ArsR) against putative MAs(III)-selective ArsR* (together indicated as "organic As") and As(III)-responsive ArsR (together indicated as "inorganic As") proteins. Black arrows highlights the cysteine pair conserved in the MAs(III) binding site of the ArsR*. For simplicity only the C-terminal region is shown. The locus tags in GenBank and JGI are: BCP1_ArsR* (JGI: Ga0035244_05158, NCBI: N505_RS21280), BCP1_ArsR (JGI: Ga0035244_05182, NCBI: N505_RS21405); *R. equi* 103S 103S_ArsR (JGI/NCBI: REQ_04560), 103S_ArsR* (JGI/NCBI: REQ_04620); *R. opacus* B4: B4_ArsR1 (JGI/NCBI: ROP_00150), B4_ArsR* (JGI/NCBI: ROP_40650), B4_ArsR2 (JGI/NCBI: ROP_29950); *R. pyridinivorans* SB3094: ArsR1 (JGI: Rpyr3094_04607; NCBI: Y013_RS15395), ArsR1* (JGI: Rpyr3094_04612; NCBI: Y013_RS15420), ArsR2* (JGI: Rpyr3094_04678, NCBI: Y013_RS25175), ArsR2 (JGI: Rpyr3094_04684, NCBI: Y013_RS25210); R. *jostii* RHA1 (JGI: RHA1_ro04133-ro04132; NCBI: RHA1_RS20100-RS20095).

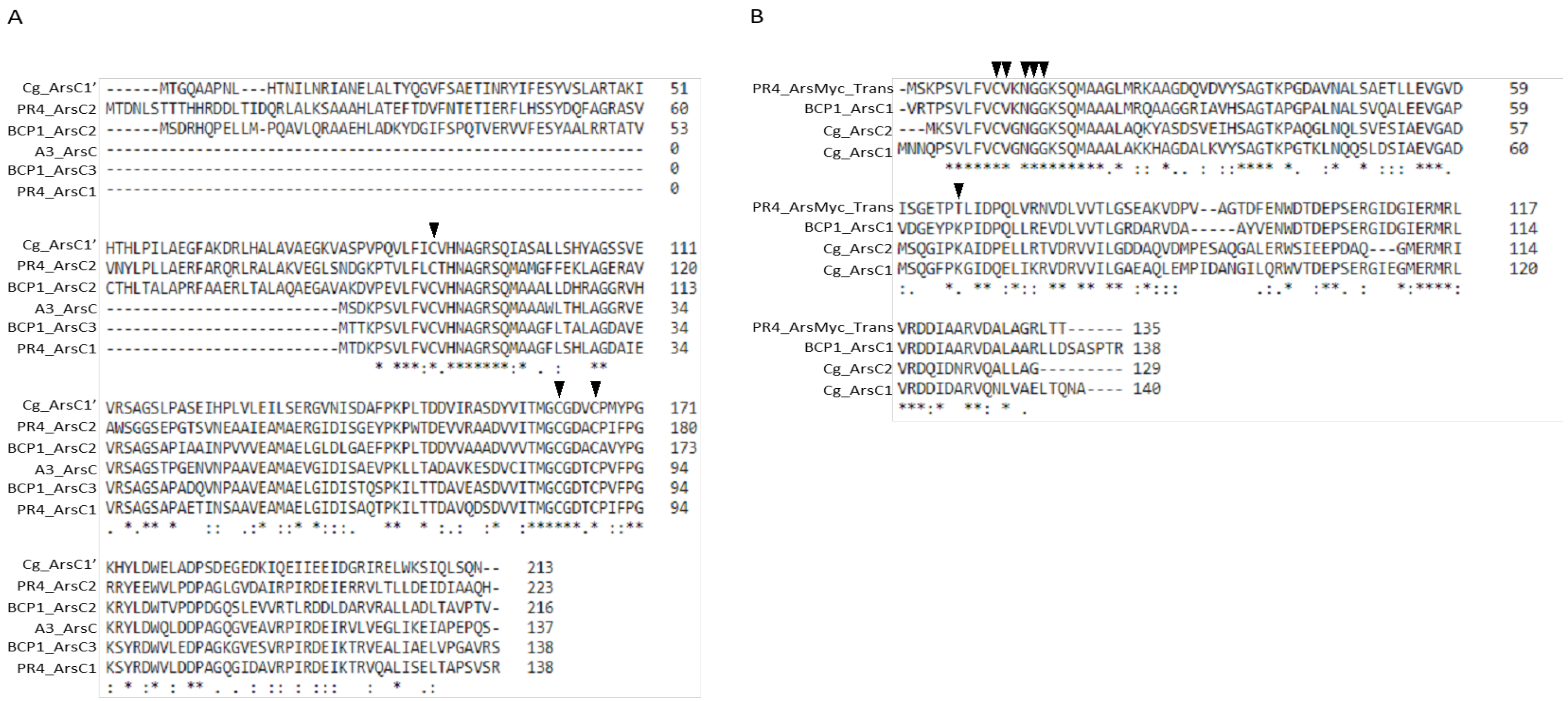

Figure S4 Multi-sequence alignment of *R. aetherivorans* BCP1 arsenate reductases BCP1_ArsC2/3 (A) and BCP1_ArsC1 (B) with the corresponding homologs from *Corynebacterium glutamicum*, *R. erythropolis* PR4 and *Streptomyces coelicolor* A3. Sequence alignment was performed with Clustal Omega (https://www.ebi.ac.uk/Tools/msa/clustalo/)

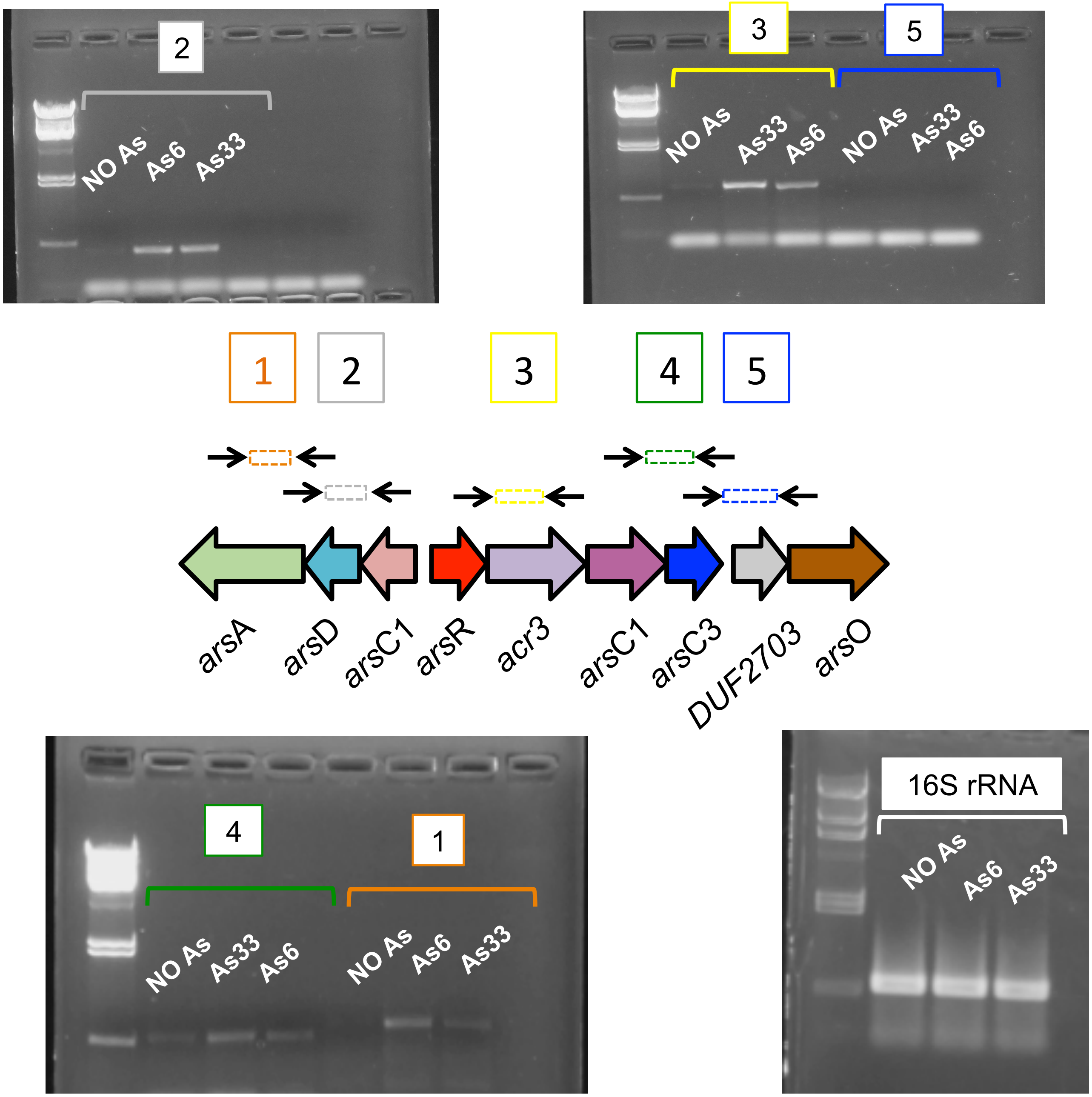

Figure S5 Analysis of *ars* genes co-transcription in *R. aetherivorans* BCP1. cDNA was retro-transcribed from mRNA extracted from BCP1 cells grown in the presence of 6mM and 33 mM As(V) (As6 or As33) or in the absence of the oxyanion (NO As). Arrows above the *ars* genes represent the position of the primers used in RT-PCR (see Supplementary Table S1) to evaluate the co-transcription of adjacent *ars* genes. The RT-PCR of 16S rRNA is also shown as reference gene.

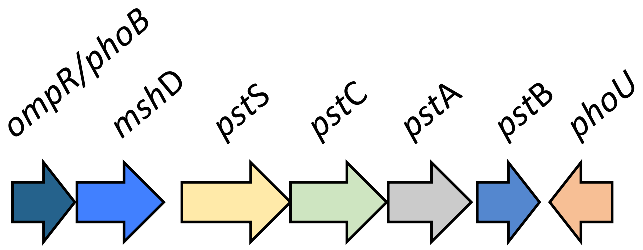

Figure S6 Organization of the *pst* genes and *mshD* gene in *R. aetherivorans* BCP1 genome. The genes displayed have JGI locus tag from Ga0035244_01310 to Ga0035244_01316, while the gene products have GenBank ID from KDE14905 to KDE14911. The predicted gene products are (in order from the left): OmpR/PhoB-type DNA-binding domain protein, mycothiol synthase MshD, phosphate transport system substrate-binding protein PstS, phosphate transport system permease protein PstC, phosphate ABC transporter PstA, phosphate transport system ATP-binding protein PstB, phosphate transport system protein PhoU.

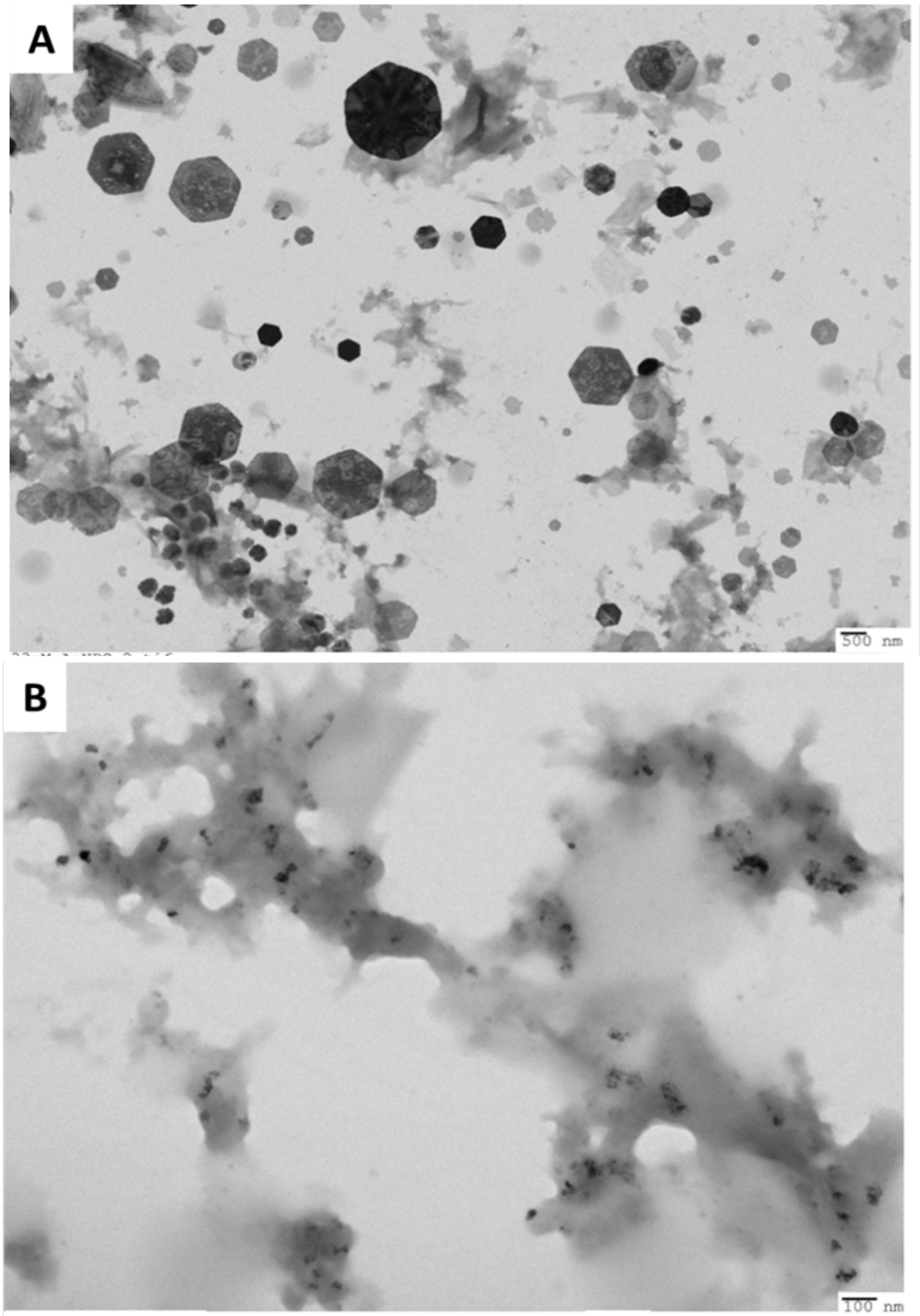

Figure S7 Transmission Electron Microscopy (TEM) images of cell extracts obtained through sonication of BCP1 cells grown for 96 hours on M9 medium and glucose 0.2% w/v as only carbon and energy source in the presence of 33 mM As(V) (Panel A) or 6 mM As(V) (Panel B). Electron dense nanoparticles are visible in the cellular extract of BCP1 cells incubated with arsenate.
